# Genotypic and Phenotypic Characterization of Antimicrobial Resistance and Virulence in *Campylobacter* spp. Isolated from Turkeys: Uncovering a Neglected Reservoir in the One Health Context

**DOI:** 10.3390/antibiotics14090935

**Published:** 2025-09-16

**Authors:** Sebastian Alexandru Popa, Viorel Herman, Khalid Ibrahim Sallam, Emil Tîrziu, Claudiu Andor, Adriana Morar, Mirela Imre, Alexandra Ban-Cucerzan, Răzvan-Tudor Pătrînjan, Alexandra Pocinoc, Kálmán Imre

**Affiliations:** 1 Department of Animal Production and Veterinary Public Health, Faculty of Veterinary Medicine, University of Life Sciences “King Mihai I”, 300645 Timisoara, Romania; emiltirziu@usvt.ro (E.T.); adrianamorar@usvt.ro (A.M.); alexandracucerzan@usvt.ro (A.B.-C.); razvan.patrinjan.fmv@usvt.ro (R.-T.P.); alexandra.pocinoc@usvt.ro (A.P.); 2 Research Institute for Biosecurity and Bioengineering, University of Life Sciences “King Mihai I”, 300645 Timisoara, Romania; 3 Department of Infectious Diseases, Faculty of Veterinary Medicine, University of Life Sciences “King Mihai I”, 300645 Timisoara, Romania; viorelherman@usvt.ro; 4 Department of Food Hygiene and Control, Faculty of Veterinary Medicine, Mansura University, Mansura 35516, Egypt; khalidsallam@mans.edu.eg; 5 Sanitary Veterinary and Food Safety Directorate of Satu Mare, 440067 Satu Mare, Romania; andor.claudiu-sm@ansvsa.ro; 6 Department of Parasitology, Faculty of Veterinary Medicine, University of Life Sciences “King Mihai I”, 300645 Timisoara, Romania; mirela.imre@usvt.ro

**Keywords:** turkey, antimicrobial resistance, public health, *Campylobacter* spp., molecular analyses, one health

## Abstract

**Background:** *Campylobacter* spp. are leading foodborne pathogens, with increasing antimicrobial resistance (AMR) posing a critical public health threat. While broiler chickens have been widely studied, turkeys represent an underexplored reservoir. The present study investigates *Campylobacter* spp. in turkeys, focusing on isolation frequency, resistance, and virulence within the context of One Health. **Methods:** A total of 182 cecal samples were collected from slaughtered turkeys in Romania. Isolation and identification of *Campylobacter jejuni* and *C. coli* followed ISO 10272-1:2017 guidelines. Antimicrobial susceptibility testing was conducted via broth microdilution, and molecular analyses using PCR targeted species identification, resistance determinants, and virulence-associated genes. **Results:** *Campylobacter* spp. were detected in 75.8% of samples, with *C. jejuni* and *C. coli* accounting for 54.3% and 45.7%, respectively. High resistance rates were observed to ciprofloxacin (89.9%) and tetracycline (85.5%), with moderate resistance to erythromycin and ertapenem. No resistance was found to gentamicin or chloramphenicol. Genotypic analyses confirmed the presence of resistance genes (e.g., *tetO*, *gyrA*—Thr-86-Ile mutation, *ermB*, *cmeB*) and widespread virulence genes (*flaA*, *cadF*, *cdtAB*, *ciaB*), supporting phenotypic results. **Conclusions**: The survey highlights turkeys as a significant but neglected source of resistant and virulent *Campylobacter* spp., with implications for food safety and public health. The convergence of AMR and virulence aspects calls for integrated surveillance and control strategies across veterinary and human health sectors, supporting the One Health strategy.

## 1. Introduction

In an increasingly interconnected world, the emergence, persistence, and spread of infectious diseases caused by microorganisms, including bacteria, viruses, and parasites, pose a significant challenge to public health, food safety, and global biosecurity [1]. Zoonotic pathogens, especially those transmissible between animals and humans, are of particular importance due to their ability to cross species barriers and spread through complex pathways involving food systems, environmental contamination, and direct or indirect transmission to humans [2,3].

The One Health concept, which emphasizes the interdependence of human, animal, and environmental health, has become essential in addressing these challenges. It calls for coordinated efforts among veterinarians, physicians, microbiologists, food producers, public health authorities, and policymakers to monitor, control, and prevent the spread of pathogens that impact multiple sectors simultaneously [4,5].

Among the various zoonotic pathogens, *Campylobacter* spp. is one of the most incriminated bacteria which cause gastroenteritis in humans not only in Europe but globally [6,7]. Infections are commonly linked to the consumption of contaminated food [8,9], poultry meat being the most identified source [10,11], and to direct or indirect contact with pets [12], livestock [13] and wild animals [14,15]. *Campylobacter jejuni* (*C. jejuni*) and *Campylobacter coli* (*C. coli*) are the most frequently isolated species from human cases [16,17] and their frequency in livestock highlights the complexity of controlling their spread [10]. The transmission nature of *Campylobacter* spp., involving animal production, food processing, environmental contamination, and consumer practices, highlights the need for a comprehensive One Health approach [18]. As such, reducing the burden of campylobacteriosis requires collaborative action across disciplines and sectors, from improving on-farm biosecurity to enhancing food hygiene and raising consumer awareness [19,20].

Another major challenge of our time is represented by the increased rates of antimicrobial resistance (AMR) especially in pathogenic microorganisms [21]. This phenomenon can make common infections increasingly difficult to manage, resulting in extended illness duration, higher healthcare costs, and a higher risk of severe outcomes or mortality [22]. AMR represents a critical concern for both human and veterinary medicine, as its emergence is strongly influenced by the widespread and, at times, inappropriate use of antibiotics across clinical, agricultural, and food production industry [23]. *Campylobacter* spp. is one of the foodborne bacteria that shows increasing resistance to important antibiotics, especially fluoroquinolones and macrolides which are drugs often used to treat infections in humans [24,25]. When bacteria become resistant, treatment options become limited, especially for vulnerable patients. The use of antimicrobials in animal farming, and inadequate diseases treatments, plays a key role in the development and spread of resistant bacterial strains. This makes *Campylobacter* spp. not only a food safety issue but also a clear example of how AMR connects human, animal, and environmental health [26].

While broiler chickens have traditionally received the most attention as a reservoir for *Campylobacter* spp., turkeys represent an equally important but often underestimated and under-researched source in the epidemiology of this pathogen [27,28,29]. As the global demand for turkey meat increases, particularly in industrialized countries, so does the importance of understanding its potential as a source of *Campylobacter* spp. transmission. Turkeys, like other poultry species, can become colonized with the aforementioned bacteria during rearing and may carry high bacterial loads without showing clinical signs of disease [30]. These bacteria can then contaminate carcasses during slaughter and processing [31], contributing to the risk of foodborne infections in humans. Despite this, surveillance and control strategies have often focused more on chickens, leaving a gap in knowledge and targeted interventions for turkeys. Given their growing role in the poultry sector, there is a clear need to investigate *Campylobacter* occurrence, antimicrobial resistance patterns, and potential risk factors specific to turkeys in order to strengthen food safety and public health efforts within a One Health framework.

Given the fact that turkeys are a source of *Campylobacter* spp. and the rising concern over antimicrobial resistance, the present study aimed to improve understanding of the public health risks associated with turkey production. The primary objectives of this research were to determine the frequency of *Campylobacter* spp. in turkeys, to assess the antimicrobial resistance profiles of the isolates, and to conduct molecular analyses for the identification of species, virulence, and resistance-associated genes. By integrating microbiological, phenotypic, and genotypic approaches, this study seeks to provide valuable data that can inform targeted interventions, surveillance programs, and biosecurity measures within the poultry industry, ultimately supporting broader One Health strategies to control *Campylobacter* spp. at the animal, human, and environment interface.

## 2. Results

### 2.1. Isolation of Campylobacter spp.

The presence of *Campylobacter* spp. was determined in a total of 138 cecal samples collected from turkeys at slaughterhouse level. Both *C. coli* and *C. jejuni* were identified among the isolates. The frequency of each species detected together with corresponding 95% confidence intervals (95% CI) are presented in Table 1.

As shown in Table 1, both *C. coli* and *C. jejuni* were identified in the examined turkey cecal samples, reflecting a high overall frequency of *Campylobacter* spp. (138/182, 75.8%) within the studied population. *C. jejuni* was the predominant species, detected in 54.3% (75/138) of samples (95% CI: 46.0–62.4), while *C. coli* was found in 45.7% (63/138; 95% CI: 37.6–54.0).

### 2.2. Antimicrobial Susceptibillity Tests Results

All confirmed *C. jejuni* (*n* = 75) and *C. coli* (*n* = 63) isolates from turkey cecal samples were tested for antimicrobial susceptibility using the broth microdilution method, following standardized procedures for minimum inhibitory concentration (MIC) determination. The testing was performed in accordance with the guidelines of the European Union and interpreted using established epidemiological cut-off values and MIC breakpoints provided by EUCAST. The results are summarized in Table 2.

Resistance to ciprofloxacin was the most frequently observed across all isolates. Specifically, 70 out of 75 *C. jejuni* isolates (93.3%) and 54 out of 63 *C. coli* isolates (85.7%) were classified as resistant to this fluoroquinolone antibiotic based on established MIC breakpoints. The high percentage in both species indicates a consistent trend of reduced susceptibility to ciprofloxacin among the tested isolates. Tetracycline resistance was also commonly detected. Among the *C. jejuni* isolates, 58 of 75 isolates (77.3%) exhibited resistance, whereas among *C. coli*, 59 of 63 isolates (93.7%) were resistant. Tetracycline was the second most affected antimicrobial agent, showing high resistance levels in both bacterial species, with a particularly higher proportion among *C. coli*.

Erythromycin resistance was observed in a lower proportion of isolates. A total of 14 *C. jejuni* isolates (18.7%) and 18 *C. coli* isolates (28.6%) were classified as resistant to this macrolide antimicrobial. This reflects a moderate level of resistance in both groups, with a slightly higher occurrence among *C. coli* isolates.

For ertapenem, 11 out of 75 *C. jejuni* isolates (14.7%) demonstrated resistance, while a lower proportion, 8 out of 63 *C. coli* isolates (12.7%), were resistant. This represents a notable resistance rate to this carbapenem, particularly among *C. coli* isolates.

No resistance was detected to chloramphenicol or gentamicin in any of the tested isolates. All *C. jejuni* and *C. coli* isolates were fully susceptible to both antimicrobial agents.

### 2.3. Correlation and Clustering Analyses of Antimicrobial Resistance in Campylobacter spp. Isolates

Pairwise associations between antibiotics were assessed to identify potential co-resistance trends. After correction for multiple testing, only the association between ciprofloxacin and tetracycline in *C. coli* remained statistically significant (r = 0.21, *p* < 0.05), indicating a weak but noteworthy link. As expected, chloramphenicol and gentamicin, to which no resistance was observed, showed no associations. Overall, these results suggest limited co-resistance in the dataset, with the ciprofloxacin–tetracycline association representing the only relevant finding.

**Figure 1:**
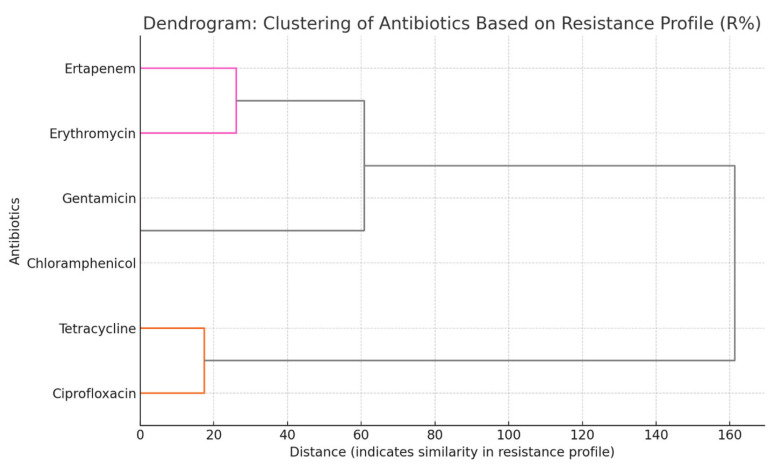
Dendrogram of antibiotic clustering based on resistance profiles (R%). Horizontal distances reflect similarity in resistance between antibiotics, with shorter distances indicating more similar resistance patterns across *C. jejuni* and *C. coli*. Colored branches show clusters of antibiotics that group below the defined distance threshold (60), suggesting potential co-resistance or similar selective pressure. Gentamicin and Chloramphenicol share identical resistance profiles (R% = 0), resulting in a zero-clustering distance; therefore, they appear on the same vertical line without visible branch separation.

To further explore similarities in resistance patterns, a hierarchical cluster analysis was performed based on the percentage of resistant *C. jejuni* and *C. coli* isolates for each antibiotic. The resulting dendrogram (Figure 1) illustrates how antibiotics group according to the similarity of their resistance rates, offering complementary insight into potential co-resistance and shared resistance mechanisms. Unlike the isolate-level correlation analysis, which evaluates binary resistance and susceptibility outcomes, clustering compares overall resistance frequencies across all isolates. Consequently, antibiotics may appear in the same cluster even when direct pairwise correlations are weak or absent.

### 2.4. Molecular Analyses

From the *Campylobacter* spp. isolates, several genes related to antimicrobial resistance and virulence were identified using PCR analysis. These include genes that may contribute to antibiotic resistance and others involved in colonization or interaction with the host. Table 3 summarizes the genes investigated, their function, and their frequency among the tested isolates.

PCR analysis revealed a high frequency of several antimicrobial resistance and virulence genes among the *Campylobacter* isolates. Among resistance genes, *gyrA* (Thr-86-Ile mutation) was detected in 90.4% of *C. jejuni* and 79.5% of *C. coli* isolates, while the *tetO* gene was nearly ubiquitous (100% in *C. jejuni*, 93.2% in *C. coli*). The *ermB* gene, screened only in erythromycin-resistant isolates, was present in all tested isolates (100%), as was the *Ery23S* (A2075G) mutation in 71.4% of *C. jejuni* and 50.0% of *C. coli*. The *cmeB* gene, associated with carbapenem resistance, was also frequently found (90.9% and 75.0%, respectively). In contrast, *catI* and *aphA-3* were not detected in any of the isolates. Regarding virulence genes, *flaA* was found in all isolates, while *cadF*, *cdtA*, *cdtB*, and *ciaB* were present in over 84% of both species. The *virB11* gene showed more variable frequency, being identified in 63.5% of *C. jejuni* and 47.7% of *C. coli* isolates.

### 2.5. Statistical Analyses Results

Statistical analysis revealed several significant differences between *C. jejuni* and *C. coli* isolates. Chi-square testing showed that resistance to tetracycline was significantly higher in *C. coli* (93.7%) compared to *C. jejuni* (77.3%) (χ^2^ = 6.12, *p* = 0.013), indicating a species-specific resistance pattern. Similarly, erythromycin resistance appears to be more frequent in *C. coli* (28.6%) than in *C. jejuni* (18.6%), although this difference was not statistically significant (χ^2^ = 2.17, *p* = 0.14). No significant differences were found in resistance to ciprofloxacin or ertapenem between the two species (*p* > 0.05). Regarding virulence genes, *cadF*, *ciaB*, and *cdtAB* were detected at high frequencies in both species, with no statistically significant interspecies variation (*p* > 0.05), except for *virB11*, which was more frequently observed in *C. jejuni* (63.5%) than in *C. coli* (47.7%), though the difference approached but did not reach statistical significance (χ^2^ = 2.63, *p* = 0.10).

## 3. Discussion

*Campylobacter* spp. are among the most important foodborne pathogens, and the increasing emergence of resistant strains represents a growing public health concern [15,32,33]. While international efforts have largely focused on broiler chickens, turkeys remain an underexplored reservoir of *Campylobacter* spp. in both scientific research and surveillance programs. In Romania, the lack of scientific data is particularly evident, with no previously published studies providing a detailed assessment of the molecular pathogenic traits or resistance determinants of *Campylobacter* bacteria isolated from turkeys. To the best of the authors’ knowledge, this is the first comprehensive study in the country to investigate the frequency of isolation, antimicrobial resistance profiles, and molecular characteristics of *Campylobacter* spp. from turkeys. These findings emphasize the need to expand monitoring and control measures in turkey production systems and support the integration of a One Health perspective that links animal, human, and environmental health in addressing foodborne threats.

In the present study, *Campylobacter* spp. were detected in 75.8% (138/182) of the examined turkey cecal samples collected at the slaughterhouse level, indicating a notably high frequency among commercially raised turkeys, in Romania. The samples originated from a slaughterhouse that processed birds supplied by farms distributed across various regions of the country, giving the results a broad national relevance. This high isolation rate supports existing evidence that poultry, especially broiler chicken [32,33,34,35,36], but also turkeys [37,38], serve as significant reservoirs of *Campylobacter* spp. Nevertheless, surveillance efforts have traditionally focused on broiler chickens.

Regarding species distribution, both *C. jejuni* and *C. coli* were detected, representing 54.3% and 45.7% of isolates, respectively. A similar proportion of *C. jejuni* and *C. coli* distribution rate, in the same matrix, has also been reported in other scientific studies [39,40]. In contrast, other research identified one species in a much higher proportion: *C. coli* [41,42] or *C. jejuni* [43]. The presence of both thermotolerant *Campylobacter* spp., indicates that turkeys efficiently host the aforementioned species, posing a significant potential route for contamination during slaughter [44,45]. This represents a very important aspect because turkey meat represents a meaningful part of poultry consumption, with Romania recording an average poultry meat intake of approximately 21.6 kg per person in 2022 [46]. At the European level, total poultry meat consumption is estimated at around 24–25 kg per capita, with turkey meat contributing significantly, particularly in countries such as Germany, Spain, and Greece [47]. These figures underline the public health importance of poultry, including turkeys in the food chain. Although they are already included in national and EU monitoring frameworks, the findings of the present study emphasize the need for more detailed investigations into turkeys, not only in Romania where data is very limited, but globally, focusing on isolation frequency, antimicrobial resistance, and molecular characteristics of *Campylobacter* spp. as part of a broader One Health strategy.

Also, the AMR profiles of the isolates were determined in the present research. Unfortunately, the antimicrobial susceptibility results revealed a concerning level of resistance among the *Campylobacter* isolates collected from turkeys. Notably, resistance to fluoroquinolones (CIP) and tetracycline (TET) was widespread, with resistance rates exceeding 85% in both *C. jejuni* and *C. coli* isolates. These findings are particularly alarming considering the critical role of these antibiotics in the treatment of human campylobacteriosis [48,49]. Resistance to erythromycin, a macrolide considered a first-line treatment option [50], was also observed in a moderate percentage of isolates, more frequently in *C. coli* (28.6%) than in *C. jejuni* (18.6%). In contrast, all isolates remained fully susceptible to gentamicin and chloramphenicol, and no phenotypic resistance was detected against these agents.

A highly significant finding was the unexpectedly high level of ertapenem resistance observed in *Campylobacter* spp., particularly in *C. jejuni* (14.6%) and *C. coli* (12.7%) isolates. While carbapenems, including ertapenem, are not used in veterinary medicine and are reserved exclusively for treating serious infections in humans, their inclusion in antimicrobial susceptibility testing for *Campylobacter* isolates from food-producing animals is mandated under EU surveillance programs. According to Commission Implementing Decision 2020/1729/EU, ertapenem is tested as part of a broader effort to detect emerging resistance to critically important antimicrobials, even in non-clinical settings [51]. This serves as an early warning mechanism to monitor potential resistance developments that could compromise human treatment options. The European Reference Laboratory for Antimicrobial Resistance (EURL-AR) has also launched initiatives such as the CarbaCamp project, which aim to establish wild-type MIC distributions and epidemiological cut-off values (ECOFFs) for carbapenems in *Campylobacter* spp. [52] Even if the resistance rates for ertapenem were low, the findings observed in this study highlight the complex antimicrobial landscape of *Campylobacter* spp. poultry, especially in turkeys, and raise important concerns about potential transmission of resistant isolates through the food chain.

In addition to phenotypic resistance profiling, molecular analyses were conducted to investigate the genetic mechanisms responsible for both antimicrobial resistance and virulence in the isolated *Campylobacter* bacteria. This genotypic characterization provides essential insight into the specific genes that may drive the observed resistance patterns and pathogenic potential. The detection of key resistance determinants, including *ermB*, *Ery23S* (A2075G mutation), and *cmeB*, was limited to isolates exhibiting phenotypic resistance to erythromycin or ertapenem, ensuring analytical relevance and methodological efficiency. Likewise, the presence of virulence-associated genes such as *cadF*, *cdtA*, *cdtB*, *ciaB*, *flaA*, and *virB11* was assessed across a representative subset of isolates to elucidate potential strain-level differences in pathogenicity. These molecular findings complement the phenotypic data and contribute to a more comprehensive understanding of the epidemiological and clinical significance of *Campylobacter* spp. circulating in turkey production systems.

In line with phenotypic antimicrobial resistance profiles, molecular analyses revealed the presence of several key AMR determinants that support and explain the resistance patterns observed in *Campylobacter* bacteria isolated from turkeys. Among phenotypically tetracycline-resistant isolates, the *tetO* gene was detected in all *C. jejuni* and in the majority of *C. coli* isolates, a well-known resistance determinant that confers ribosomal protection and is commonly associated with high tetracycline resistance rates in *Campylobacter* spp. [53,54,55]. Similarly, ciprofloxacin-resistant isolates showed a strong correlation with the presence of the *gyrA* Thr-86-Ile point mutation in the quinolone resistance–determining region (QRDR), which are known to reduce fluoroquinolone binding and contribute to high-level resistance [56]. For erythromycin, the detection of the *ermB* gene which is a gene encoding a methyltransferase that modifies the 23S rRNA target site, was confirmed in all isolates exhibiting phenotypic resistance [57]. Furthermore, the *Ery23S* (A2075G) point mutation and the efflux pump gene *cmeB* were also identified in subsets of resistant isolates, suggesting the involvement of multiple mechanisms in conferring macrolide resistance [58]. Importantly, the genotypic data fully supported the phenotypic AST results, with resistance genes detected exclusively in isolates that had shown phenotypic resistance to the corresponding antibiotics. This concordance strengthens the reliability of the resistance screening and highlights the utility of combining phenotypic and molecular approaches to gain a comprehensive understanding of resistance dissemination in *Campylobacter* spp. from poultry. The absence of resistance genes *aphA-3* and *catI* in all isolates aligned with the total phenotypic susceptibility to gentamicin and chloramphenicol, respectively, further validating the correlation between genetic markers and observed antimicrobial behavior.

Although phenotypic resistance and the presence of corresponding genes showed a high level of agreement, a small number of discrepancies were observed. Resistant isolates without the targeted gene may possess alternative mechanisms, such as efflux pump overexpression or mutations in untested genomic regions, and in some cases the genetic determinant may have low penetrance [49,58]. Conversely, the presence of a resistance gene in susceptible isolates may be due to non-functional variants, low expression under the test conditions, or regulatory influences limiting gene activity [58,59]. Future work will incorporate whole-genome sequencing of representative isolates to provide a more complete understanding of these discordances and the diversity of underlying resistance mechanisms.

While detecting antimicrobial resistance genes offers valuable insight into the therapeutic challenges posed by *Campylobacter* spp. in turkey production, understanding the genetic factors underlying pathogenic potential is equally important. Virulence-associated genes are crucial for host colonization, immune evasion, and tissue damage. Therefore, alongside resistance profiling, this study examined well-characterized virulence determinants to better understand mechanisms supporting host colonization and potential zoonotic transmission.

Successful colonization of the host gastrointestinal tract represents a critical initial step in *Campylobacter* pathogenesis, with motility playing a main role in this process. The bacterial flagellar apparatus, primarily composed of the structural proteins *flaA* and *flaB* which are encoded by adjacent genes, facilitates movement through the intestinal mucus layer [60]. In the present study, the *flaA* gene, which encodes a major structural component of the *Campylobacter* flagellum, was detected in 100% of the tested isolates, highlighting its essential role in bacterial motility, colonization, and initial host interaction. The results are in accordance with other studies from European level [61,62,63,64] and worldwide [58,61,65]. The universal presence of this gene suggests that flagella-mediated motility remains a fundamental virulence mechanism across *Campylobacter* circulating in turkeys, potentially contributing to effective mucosal penetration and persistence within the gastrointestinal tract.

The *cadF* gene was also detected in the majority of *Campylobacter* spp. isolates (89.6%) obtained from turkeys, consistent with its role as a key virulence factor involved in host adhesion. The *cadF* gene encodes a fibronectin-binding outer membrane protein that facilitates the attachment of bacterial cells to the intestinal epithelium, representing a critical first step in colonization and infection [66]. Its high frequency among poultry isolates, including those from turkeys, supports the idea that *cadF* is highly conserved and under selective pressure due to its importance in maintaining colonization efficiency within the host gastrointestinal tract. The presence of this gene not only reflects the pathogenic potential of these isolates but also suggests their ability to persist in poultry reservoirs and possibly contribute to human infection through the food chain [67].

Among the virulence factors assessed in this study, particular attention was given to the *cdtA* and *cdtB* genes, which form part of the *cdtABC* operon encoding the cytolethal distending toxin (CDT) [68]. These genes were identified in almost all isolates from turkeys. As structural and functional components of CDT, CdtA facilitates host cell binding and toxin delivery, while CdtB exerts DNase-like activity that leads to DNA damage and subsequent cell cycle arrest. The presence of these genes in a considerable proportion of isolates indicates a conserved virulence mechanism that may contribute to intestinal epithelial damage and inflammation in infected hosts [69]. Their detection was in accordance with other studies all over the world [58,70,71,72] which provide further evidence regarded potential of zoonotic transmission and reinforces the importance of monitoring CDT-related genes in foodborne *Campylobacter* [73].

To evaluate the invasive potential of *Campylobacter* isolates from turkeys, the presence of two key invasion-related genes, *virB11* and *ciaB*, was examined. The *ciaB* gene, which encodes a *Campylobacter* invasion antigen, was detected in a high proportion of isolates (89.6%), consistent with its recognized role in facilitating bacterial internalization into host epithelial cells. This gene is part of the *Campylobacter* flagellum-dependent secretion system and has been shown to be actively expressed during host–pathogen interactions, promoting cytoskeletal rearrangements and enhancing invasion efficiency [74]. The present study results are in accordance with other research conducted in Brazil [75], South Korea [76], Tunisia [58] and Poland [61]. In contrast, the *virB11* gene, part of the type IV secretion system typically found on plasmids such as pVir, was identified in a smaller subset of isolates (56.3%). Although not universally present, *virB11* has been associated with increased invasiveness and may represent an accessory virulence factor in certain *C. jejuni* strains [62]. The differential frequency of these two genes in turkey-derived isolates supports the notion that while *ciaB* represents a core component of *Campylobacter* spp. invasive strategy, *virB11* may contribute to enhanced pathogenic potential in select strains carrying virulence plasmids [77].

Despite the valuable insights provided by the present research into the resistance and virulence profiles of turkey-derived *Campylobacter* spp., certain limitations should be considered. Due to technical and financial constraints, molecular testing was performed on a statistically representative subset of isolates rather than the entire collection. While this approach ensured robust and scientifically valid conclusions, it may have limited the detection of rare resistance genes or atypical genotype–phenotype patterns. Also, a limitation of the present study is that, due to the selection of a limited number of morphologically typical colonies per sample for species identification, potential coexistence of *C. jejuni* and *C. coli* within the same cecal sample could not be detected. This approach, while consistent with standard protocols, may have underestimated mixed-species colonization rates, which have been occasionally reported in poultry.

## 4. Materials and Methods

### 4.1. Study Design and Samples Collection

The present study was conducted to investigate the frequency of isolation, antimicrobial resistance and molecular characteristics of *Campylobacter* spp. isolated from turkeys in Romania. A total of 182 cecal samples were randomly collected from turkeys during slaughter process at a commercial slaughterhouse specialized in processing turkeys originating from farms distributed across all regions of Romania. This facility receives animals from a wide geographic range, making it a representative point for national-level sampling. The samples were collected monthly over 2024 and first months of 2025, as part of the investigation into *Campylobacter* spp. isolation frequency and antimicrobial resistance in the turkey sector. The turkeys were slaughtered at approximately 14 to 20 weeks of life, depending on sex and production type, which reflects standard commercial practices in the country. All samples were obtained aseptically during the evisceration stage, placed in sterile containers, and immediately stored and transported under refrigerated conditions (~4 °C) to the Laboratory of Microbiology Risk Surveillance at the Faculty of Veterinary Medicine in Timișoara. Microbiological processing was performed within 24 h of collection to ensure sample integrity and reliability of results.

### 4.2. Isolation and Identification of Campylobacter spp.

The detection and isolation of *Campylobacter* spp. from turkey cecal samples were performed according to ISO 10272-1:2017 [78] guidelines, using direct plating method. Firstly, a small incision was made in the cecum, and a 10 µL loopful of intestinal content was aseptically streaked onto two selective agar media as follows: modified charcoal cefoperazone deoxycholate agar (mCCDA; Oxoid Ltd., Basingstoke, UK) and Butzler agar (Oxoid Ltd., Basingstoke, UK).

All inoculated plates were incubated at 41.5 °C for 44 h under microaerobic conditions (5% O_2_, 10% CO_2_, 85% N_2_), using anaerobic jars and gas-generating microaerobic kits (Thermo Scientific™, Waltham, MA, USA). Following incubation, colonies with typical morphology—grayish with a metallic sheen and moist appearance on mCCDA; grayish to brown-orange on Butzler agar—were selected for subculturing onto Columbia blood agar (Oxoid Ltd., Basingstoke, UK) to obtain pure isolates. These plates were incubated under identical conditions for an additional 44 h. Pure colonies were then subjected to a series of phenotypic and biochemical tests. Gram staining was performed to confirm characteristic cell morphology (curved, spiral, or S-shaped forms), while oxidase and catalase activity were assessed using standard biochemical tests (Oxoid Ltd.). Growth inhibition at 25 °C under aerobic conditions was also evaluated to verify microaerophilic requirements. Additionally, the DrySpot agglutination test (Oxoid Limited, Basingstoke, United Kingdom) was used for further confirmation of *Campylobacter* spp. identity. Reference strains *Campylobacter jejuni* ATCC 33291 and *Campylobacter coli* ATCC 43478 were used as positive controls throughout the procedure. The nuclease free water was used as the negative control in each PCR run to validate the assay.

### 4.3. Antimicrobial Susceptibiliy Tests

In accordance with the guidelines established by EU Decision No. 652/2013 [79], all *Campylobacter* spp. isolated microorganisms in the present research were subjected to antimicrobial susceptibility testing (AST) using the broth microdilution method for minimum inhibitory concentration (MIC) determination which is considered the gold standard for assessing antimicrobial resistance and provides quantitative data that support surveillance and risk assessment within the One Health approach. Pure colonies isolated on Columbia blood agar (Oxoid Ltd., Basingstoke, UK) were prepared in Tryptic Soy Broth (Becton, Dickinson and Company, Sparks, MD, USA), with the bacterial suspension adjusted to a turbidity equivalent to a 0.5 McFarland standard, ensuring consistent inoculum density across all tests. The standardized inoculum was then transferred into Müller–Hinton broth (Oxoid Ltd., Basingstoke, UK) supplemented with 2.5–5% lysed horse blood to support the growth of fastidious *Campylobacter* strains.

The prepared solutions were dispensed into EUCAMP microdilution plates (Sensititre™, Thermo Scientific™, Waltham, MA, USA), which contain predefined concentration ranges of antimicrobial agents recommended for *Campylobacter* spp. susceptibility testing. The antimicrobial susceptibility profiles of all *C. jejuni* and *C. coli* isolates were determined against a panel of six antimicrobial agents representing six major antibiotic classes: fluoroquinolone (ciprofloxacin—CIP; 0.125–32 µg/mL), amphenicols (chloramphenicol—CHL; 2–64 µg/mL), macrolide (erythromycin—ERY; 1–512 µg/mL), aminoglycoside (gentamicin—GEN; 0.25–16 µg/mL), tetracycline (tetracycline—TET; 0.5–64 µg/mL) and carbapenem β-lactam (ertapenem—ERT; 0.125–4 µg/mL). The plates were incubated under microaerobic conditions at 41.5 °C for 48 h. The MICs were determined as the lowest concentration of each antimicrobial that visibly inhibited bacterial growth. This procedure allows for precise evaluation of resistance levels and supports comparability with data collected through national and EU-wide antimicrobial resistance monitoring programs [80].

### 4.4. Molecular Analyses

All isolates confirmed through microbiological examination were subjected to molecular analysis to verify their identity at both genus and species levels, as well as to assess the presence of virulence-associated genes and antimicrobial resistance determinants through genotypic screening. The sequences, targets, and references for all primers utilized in this study for species identification, virulence gene detection, and resistance gene analysis are listed in Supplementary Table S1.

Genomic DNA was extracted using the ISOLATE II Genomic DNA Kit (BIOLINE^®^, UK Ltd., London, UK), following the manufacturer’s instructions. Genus-level confirmation of *Campylobacter* spp. was achieved through a conventional polymerase chain reaction (PCR) targeting the *23S rRNA* gene (~650 bp). Amplification was carried out using the primer pair 23SF (5′-TATACCGGTAAGGAGTGCTGGAG-3′) and 23SR (5′-ATCAATTAACCTTCGAGCACCG-3′), following the thermal cycling conditions described by Wang et al. [81].

Species identification was subsequently performed by targeting specific genes unique to *C. jejuni* and *C. coli*. For the confirmation of *C. jejuni*, PCR amplification of the *hipO* gene (~323 bp) was employed using the primers hipO-F (5′-ACTTCTTTATTGCTTGCTGC-3′) and hipO-R (5′-GCCACAACAAGTAAAGAAGC-3′), as previously described by Wang et al. [81]. For the detection of *C. coli*, a PCR assay targeting the *glyA* gene (~133 bp) was used, with primers glyA-F (5′-CATATTGTAAAACCAAAGCTTATCGG-3′) and glyA-R (5′-AGTCCAGCAATGTGTGCAATG-3′), in accordance with the protocol outlined by LaGier, M.J [82].

For the genes associated with antimicrobial resistance [*gyrA* QRDR mutation (Thr-86-Ile)—for ciprofloxacin, *catI*—chloramphenicol, *ermB* and *Ery23S*—erythromycin, *cmeB*—ertapenem, *aphA-3*—gentamicin, *tet(O)*—tetracycline] and virulence (*cadF* and *flaA* for *Campylobacter* adhesion genes; and *virB11*, *ciaB*, *cdtA*, and *cdtB* invasion genes) the PCR was performed using specific primers and following the annealing conditions as previously described by several authors [63,70,71,83,84,85,86,87,88,89,90,91]; Supplementary Table S1. For isolates subjected to *gyrA* screening, mutation analysis was additionally performed to detect fluoroquinolone resistance–associated mutations within the quinolone resistance-determining region, with particular focus on the Thr-86-Ile substitution, as described by Zirnstein et al. [83].

The PCR products were analyzed via horizontal gel electrophoresis in a 1.5% agarose matrix, stained with RedSafe™ nucleic acid dye (iNtRON Biotechnology, Gyeonggi-do, Republic of Korea). A 100 bp DNA ladder (BIOLINE^®^, UK Ltd., London, UK) was used as a molecular weight reference in the first lane. Visualization of DNA bands was performed under ultraviolet light using a UVP^®^ gel documentation system.

### 4.5. Selection of Representative Isolate Subset for Molecular Characterization

In the context of increasing concern regarding AMR and virulence evolution in *Campylobacter* species, understanding the genetic determinants behind these phenotypic aspects is essential for developing effective surveillance and control strategies in animal production systems. However, given the technically and financially demanding nature of molecular testing, in the present study a representative sampling strategy to characterize key resistance and virulence genes in a scientifically justified subset of isolates was adopted. Molecular characterization targeted several resistance and virulence genes with previously reported detected frequency ranging from 59% to 100%. To ensure that the analysis was both representative and statistically correct, minimum number of isolates required for gene prevalence estimation using the standard formula for proportions was assessed. Assuming a 95% confidence level and a conservative prevalence estimate of 50% (to account for the gene with the lowest frequency), with a ±10% margin of error, the minimum required sample size was determined to be 96 isolates. Proportional stratified sampling was then applied to preserve the natural species distribution, resulting in the inclusion of 52 *C. jejuni* and 44 *C. coli* isolates for molecular analysis. This approach ensured valid extrapolation of genetic findings while addressing the practical limitations of full-population genotyping [92,93].

### 4.6. Statistical Analyses

All statistical analyses were carried out using GraphPad software, version 10.0.2 (Boston, MA, USA). The prevalence of *Campylobacter* spp. and the frequency of antimicrobial resistance were expressed as percentages, accompanied by 95% confidence intervals (CI). Differences in the distribution of *Campylobacter* species (*C. jejuni* vs. *C. coli*) as well as variations in resistance rates across the tested antimicrobials were analyzed using the chi-square (χ^2^) test for categorical variables. A *p*-value of less than 0.05 was considered statistically significant.

In addition to prevalence comparisons, pairwise phi coefficients were calculated between binary resistance outcomes. Two-sided *p*-values were obtained using the standard *t*-test for Pearson correlation, and 95% CI were derived via Fisher’s z transformation. To account for multiple testing, *p*-values were adjusted using the Benjamini–Hochberg method (one family per species; *m = k*(*k* − 1)/2 tests, where *k* represents the number of antibiotics).

### 4.7. Bioethics Commission Approval

The present study was approved by the Bioethics Commission of University of Life Sciences “King Mihai I” from Timișoara, Romania, in accordance with national and European regulations on animal welfare and research ethics (Approval No. 473). The commission confirmed that no live animals were used in this study, as all biological samples (cecal) were collected post-mortem at the slaughterhouse level by trained personnel. The sampling procedures complied with applicable EU legislation governing the humane treatment of animals during slaughter, including Regulation (EC) No 1099/2009 on the protection of animals at the time of killing. As such, the study did not involve any procedures that would raise ethical concerns regarding animal experimentation.

## 5. Conclusions

This study offers a comprehensive characterization of *C. jejuni* and *C. coli* isolates from turkeys, providing essential insight into an underexplored reservoir within the poultry production chain. The integration of phenotypic and genotypic data revealed a high level of agreement, with all phenotypically resistant isolates carrying corresponding antimicrobial resistance genes, including *tetO*, *gyrA* (Thr-86-Ile mutation), *ermB*, *Ery23S*, and *cmeB*. Concurrently, the widespread detection of core virulence genes such as *cadF*, *flaA*, *ciaB*, and *cdtAB*, alongside the sporadic presence of *virB11*, highlights the pathogenic potential of these isolates and their possible role in zoonotic transmission. While *Campylobacter* in chickens has been extensively studied, molecular data on turkey isolates remain limited, making these findings particularly relevant. Importantly, the convergence of resistance and virulence traits in *Campylobacter* isolates from turkeys underscores the need for integrated, cross-sectoral monitoring under the One Health framework, recognizing the dynamic interplay between animal production, food safety, and human health.

Future research should expand on these findings by employing whole-genome sequencing to better elucidate the evolution and transmission dynamics of resistance and virulence genes. Moreover, longitudinal studies across the turkey production chain, from farms to processing facilities, would help identify critical control points and guide more effective biosecurity and antimicrobial stewardship strategies. Such efforts will be essential to mitigate zoonotic risks and safeguard the efficacy of key antimicrobials for both veterinary and human medicine.

In light of the molecular and phenotypic evidence presented, this study brings renewed attention to turkeys as an underappreciated and neglected reservoir of *Campylobacter* spp., highlighting a critical surveillance gap that must be addressed within the broader One Health framework.

## Figures and Tables

**Table 1:** Isolation frequency of *C. coli* and *C. jejuni* in turkey cecal samples.

Origin	*Campylobacter* spp.	No. of Positive/ No. of Samples	Prevalence, % [95% CI]
Slaughterhouse	*C. coli*	63/138	45.7 [37.6–54.0]
	*C. jejuni*	75/138	54.3 [46.0–62.4]

**Table 2:** Distribution of antimicrobial resistance among *Campylobacter* spp. isolates from turkeys.

Antimicrobial		Cut Off Values	MIC Breakpoint (µg/mL)		No. of Resistant Isolates/Total Investigated (%)	
Class	Agent		S	R	*C. jejuni*	*C. coli*
**Fluoroquinolone**	Ciprofloxacin	0.5	0.125	32	70/75 (93.3)	54/63 (85.7)
**Amphenicols**	Chloramphenicol	16	2	64	0/75 (0)	0/63 (0)-
**Macrolide**	Erythromycin	8 (4) ^a^	1	512	14/75(18.7)	18/63 (28.6)
**Carbapenem**	Ertapenem	0.5	0.125	4	11/75 (14.7)	8/63 (12.7)
**Aminoglycoside**	Gentamicin	2	0.25	16	0/75 (0)	0/63 (0)-
**Tetracycline**	Tetracycline	2 (1) ^a^	0.5	64	58/75 (77.3)	59/ 63 (93.7)

Legend: MIC—minimum inhibitory concentration; S—susceptible; R—resistant; ^a^—the MIC values were different between *C. jejuni* and *C. coli*.

**Table 3:** Resistance and virulence genes detected in *Campylobacter* spp. isolates.

Gene Function	Determinants	*C. jejuni* % (p/N)	*C. coli* % (p/N)	Total % (p/N)
Resistance	*gyrA* (Thr-86-Ile mutation)	90.4 (47/52)	81.8 (36/44)	86.5 (83/96)
	*catI*	0 (0/52)	0 (0/44)	0 (0/96)
	*ermB*	100 (14/14) ^a^	100 (18/18) ^b^	100 (32/32)
	*Ery23S* (A2075G)	71.4 (10/14) ^a^	50.0 (9/18) ^b^	59.4 (19/32)
	*cmeB*	90.9 (10/11) ^c^	75.0 (6/8) ^d^	84.2 (16/19)
	*aphA-3*	0 (0/52)	0 (0/44)	0 (0/96)
	*tetO*	100 (52/52)	93.2 (41/44)	96.9 (93/96)
Virulence	*cadF*	94.2 (49/52)	84.1 (37/44)	89.6 (86/96)
	*cdtA*	96.2 (50/52)	84.1 (37/44)	90.6 (87/96)
	*cdtB*	92.3 (48/52)	90.9 (40/44)	91.7 (88/96)
	*ciaB*	90.4 (47/52)	88.6 (39/44)	89.6 (86/96)
	*flaA*	100 (52/52)	100 (44/44)	100 (96/96)
	*virB11*	63.5 (33/52)	47.7 (21/44)	56.3 (54/96)

Legend: p—positive samples; N—total number of tested isolates; ^a, b, c, d^—molecular screening for erythromycin (*ermB*, *Ery23S*—A2075G) and ertapenem (*cmeB*) resistance determinants was performed only on isolates that showed phenotypic resistance to aforementioned antimicrobials, as these genes are specifically associated with macrolide and carbapenem resistance mechanisms.

## Data Availability

Data are contained within the article.

## Supplementary Materials

The following supporting information can be downloaded at: https://www.mdpi.com/article/10.3390/antibiotics14090935/s1, Table S1: PCR primers and conditions for detection of species, resistance, and virulence genes.
