# Supplementary material for: Genotypic and Phenotypic Characterization of Antimicrobial Resistance and Virulence in Campylobacter spp. Isolated from Turkeys: Uncovering a Neglected Reservoir in the One Health Context
Source: Antibiotics (Basel). 2025 Sep 16;14(9):935. doi: 10.3390/antibiotics14090935 (PMC12466818; doi:10.3390/antibiotics14090935)
Supplement: Supplementary file 1 [file antibiotics-14-00935-s001.zip › antibiotics-3771967-supplementary.pdf]

Supplementary Table S1

PCR primers and conditions for detection of species, resistance, and virulence genes.

Target Purpose	Agent	Targeted gene	Sequences (5′–3′)	Product size (bp)	Annealing temperature	Reference
**AMR**	Ciprofloxacin	*gyrA*	ATTTTTAGCAAAGATTCTGAT<br>CCATAAATTATTCCACCTGT	673	50°C	[83]
	Chloramphenicol	*catI*	GCATCATTTATCCTCC<br>CATCCCGAGCTTTAAA	621	56°C	[84]
	Erythromycin	*ermB*	GGGCATTTAACGAAACTGG<br>CTGTGGTATGGCGGGTAAGT	421	52°C	[85]
		Ery23S (A2075G)	GTAAACGGCCGTAACTA<br>GACCGAACTGTCTCACGACG	714	28 cycles of 90 °C for 10 s, 50 °C for 5 s, and 60 °C for 4 min	[86]
	Ertapenem	*cmeB*	AGGCGGTTTTGAAATGTATGTT<br>TGTGCCGCTGGGAAAAG	444	50°C	[87]
	Gentamycin (0)	*aphA-3*	TGCGTAAAAGATACGGAAG<br>CAATCAGGCTTGATCCCC	701	54°C	[88]
	Tetracycline	*tetO*	GCGTTTTGTTTATGTGCG<br>ATGGACAACCCGACAGAAG	559	53°C	[89]
**Virulence**	*Campylobacter* adhesion genes	cadF	TTGAAGGTAATTTAGATATG<br>CTAATACCTAAAGTTGAAAC	400	45°C	[90]
		flaA	ATGGGATTTCGTATTAACAC<br>CTGTAGTAATCTTAAAACATTTTG	1713	55°C	[91]
	Campylobacter invasion gene	*virB11*	TCTTGTGAGTTGCCTTACCCCTTTT<br>CCTGCGTGTCCTGTGTTATTTACCC	494	48°C	[70]
		ciaB	TGCGAGATTTTTCGAGAATG<br>TGCCCGCCTTAGAACTTACA	527	57°C	[71]
		cdtA	CCTTGTGATGCAAGCAATC<br>ACACTCCATTTGCTTTCTG	370	55°C	[72]
		cdtB	GTTAAAATCCCCTGCTATCAACCA<br>GTTGGCACTTGGAATTTGCAAGGC	495	51°C	[63]
